# A Replication Study of the Association between Rheumatoid Arthritis and Deletion of the Late Cornified Envelope Genes *LCE3B* and *LCE3C*

**DOI:** 10.1371/journal.pone.0032045

**Published:** 2012-02-23

**Authors:** Judith G. M. Bergboer, Maša Umićević-Mirkov, Jaap Fransen, Martin den Heijer, Barbara Franke, Piet L. C. M. van Riel, Joost Schalkwijk, Marieke J. H. Coenen, on behalf of the Nijmegen Biomedical Study principal investigators

**Affiliations:** 1 Department of Dermatology, Radboud University Nijmegen Medical Centre, Nijmegen Centre for Molecular Life Sciences, Nijmegen, The Netherlands; 2 Department of Human Genetics, Radboud University Nijmegen Medical Centre, Nijmegen, The Netherlands; 3 Department of Rheumatology, Radboud University Nijmegen Medical Centre, Nijmegen, The Netherlands; 4 Department of Endocrinology and Department of Epidemiology and Biostatistics, Radboud University Nijmegen Medical Centre, Nijmegen, The Netherlands; Brigham Young University, United States of America

## Abstract

**Objective:**

Two recent studies, in a Spanish and a Chinese population, point to an association between rheumatoid arthritis (RA) risk and the deletion of *the Late Cornified Envelope* (*LCE*) *3B* and *3C* genes (*LCE3C_LCE3B*-del), a known risk factor for psoriasis. We aimed to replicate these studies in a large Dutch cohort.

**Methods:**

1039 RA cases and 759 controls were genotyped for *LCE3C_LCE3B*-del. Association analysis was performed for the complete cohort and after stratification for the serologic markers anti-cyclic citrullinated peptide and rheumatoid factor. A meta-analysis was performed combining our data with the Spanish and Chinese datasets, resulting in an analysis including 2466 RA cases and 2438 controls.

**Results:**

In the Dutch cohort we did not observe a significant association of *LCE3C_LCE3B*-del (p = 0.093) with RA risk. A stratified analysis for the serologic positive and negative group did not show an association between the genetic variant and disease risk, either. The meta-analysis, however, confirmed a significant association (p<0.0001, OR = 1.31, 95% confidence interval 1.16–1.47).

**Conclusion:**

Our meta-analysis confirms the association of the *LCE3* deletion with RA, suggesting that *LCE3C_LCE3B*-del is a common risk factor for (auto)immune diseases.

## Introduction

Rheumatoid arthritis (RA) is a chronic inflammatory joint disease affecting approximately 1% of the population. Although the causes of the disease are largely unknown, both genetic and environmental factors seem to contribute. Thus far, genome-wide association studies have revealed more than 30 risk loci explaining 16% of the disease variance.

The copy number of DNA segments in the human genome varies in normal individuals and most likely this diversity is responsible for a significant proportion of phenotypic variation. Not much is known about the role of copy number variation (CNV) in the etiology of RA, but relevance of these common genetic variants is expected. A small number of candidate gene studies have indicated that CNVs in Chemokine ligand 3-like 1 (*CCL3L1*), Fc gamma receptor 3B (*FCGR3B*) and pre-B lymphocyte 1 (*VPREB1*) are associated with RA disease susceptibility [1], [2], [3]. Using a genome-wide approach the Welcome Trust Case Control Consortium (WTCCC) reported that CNVs in the HLA region are significantly associated with the development of RA [4]. More recently using the WTCCC dataset, 11 rare copy number variable regions associated with RA were identified [5]. These regions included genes that could be directly linked to the development of RA. Another potential candidate CNV is the deletion of two genes of the *Late Cornified Envelope* gene cluster, *LCE3B* and *LCE3C* (*LCE3C_LCE3B*-del), which was recently found associated with psoriasis in several populations [6], as was rs4112788, a single nucleotide polymorphism (SNP) in high linkage disequilibrium with the deletion [7]. The observation that the risk of RA is increased in the offspring of individuals affected with other autoimmune disorders, including psoriasis, prompted Docampo and colleagues [8] to investigate the association between the *LCE3C_LCE3B*-del and RA in two independent Spanish cohorts. In their study an association was found between RA and homozygosity for *LCE3C_LCE3B*-del. Recently it has been demonstrated that the deletion is also associated with RA susceptibility in a non-Caucasian (Chinese-Han) population [9]. However, they were unable to confirm the Spanish results that the association between *LCE3C_LCE3B*-del and RA risk was restricted to anti-CCP-positive patients. We sought to replicate these studies in a similarly powered Dutch cohort. A total of 1039 RA cases and 759 controls were genotyped for *LCE3C_LCE3B*-del, and we investigated whether this genetic variant is associated with RA. Additionally, we combined the Spanish, Chinese and Dutch data in a meta-analysis.

## Results

An overview of the demographics of the patients and controls used in this study are presented in Table 1. Table 2 provides the results of the association analysis for the *LCE3C_LCE3B*-del and RA risk in the Dutch cohort. There were 1039 RA patients and 779 controls successfully genotyped for *LCE3C_LCE3B*-del. The *LCE3C_LCE3B*-del and the linked SNP rs4112788 were in near-perfect linkage disequilibrium (LD) with each other (r^2^ = 0.928 and D′ = 0.984), confirming that *LCE3C_LCE3B*-del was genotyped accurately.

**Table 1:** Demographics of the rheumatoid arthritis cohort.

	RA patients	Controls
number	1043	779
gender (% female)	693 (66.4)	422 (54.2)
age^#^	55.0±12.7	56.3±16.8
age at disease onset^##^	45.3±13.3	
RF positive^###^	736 (77.3)	
anti-CCP positive^####^	286 (63.4)	

Numbers are depicted as n(%) or mean ± standard deviation. Data available for ^#^686, ^##^717, ^###^952, ^####^451 patients. RA: rheumatoid arthritis; CCP: anti-cyclic citrullinated peptide; RF: rheumatoid factor.

**Table 2:** No association of the *LCE3C_LCE3B* deletion with RA in the Dutch cohort.

	Cohort									
	All		All CCP−		All CCP+		All RF−		All RF+	
***LCE3C_LCE3B*** ** deletion**										
Patients										
no del/no del	149	(14.3)	18	(10.9)	43	(15.0)	24	(11.1)	112	(15.2)
del/no del	460	(44.3)	85	(51.5)	130	(45.5)	96	(44.4)	326	(44.3)
del/del	430	(41.4)	62	(37.6)	113	(39.5)	96	(44.4)	298	(40.5)
Controls										
no del/no del	118	(15.1)	118	(15.1)	118	(15.1)	118	(15.1)	118	(15.1)
del/no del	369	(47.4)	369	(47.4)	369	(47.4)	369	(47.4)	369	(47.4)
del/del	292	(37.5)	292	(37.5)	292	(37.5)	292	(37.5)	292	(37.5)
Recessive *P*	0.093		0.982		0.546		0.064		0.231	
OR (95% CI)	1.18 (0.97–1.43)		1.00 (0.71–1.42)		1.09 (0.83–1.44)		1.33 (0.98–1.81)		1.14 (0.92–1.40)	
Codominant *P*	0.242		0.339		0.822		0.116		0.436	
OR (95% CI)	0.99 (0.75–1.30) (del/no del)		1.51 (0.87–2.62) (del/no del)		0.97 (0.65–1.44) (del/no del)		1.28 (0.78–2.09) (del/no del)		0.93 (0.69–1.26) (del/no del)	
	1.17 (0.88–1.55) (del/del)		1.39 (0.79–2.45) (del/del)		1.06 (0.70–1.60) (del/del)		1.62 (0.99–2.65) (del/del)		1.08 (0.79–1.46) (del/del)	

*LCE3C_LCE3B* deletion: deletion of the *LCE3B* and *LCE3C* gene; del: deletion; CCP−: patients negative for anti cyclic citrullinated peptide; CCP+: patients positive for anti-cyclic citrullinated peptide; RF−: patients negative for rheumatoid factor; RF+ patients positive for rheumatoid factor. OR: odds ratio; 95% CI: 95% confidence interval.

In the Dutch cohort, we did not find evidence for an association between *LCE3C_LCE3B*-del and RA, at the genotypic (p = 0.093, OR 1.18, 95% confidence interval (95%CI): 0.97–1.43 (Table 2)) nor the allelic level (p = 0.147, OR 1.11 95%CI: 0.97–1.27 (data not shown)). It has been hypothesized that the *LCE3C_LCE3B*-del might result in a defective skin barrier repair leading to an abnormal or increased exposure to environmental antigens. This could lead to an earlier age of disease onset in carriers of *LCE3C_LCE3B*-del compared to non-carriers. An analysis for age of onset, however, did not show association with the *LCE3C*_*LCE3B*-del status (p = 0.25, t-test).

Docampo *et al.* [8] demonstrated that *LCE3C_LCE3B*-del showed the strongest association with RA in anti-CCP and rheumatoid factor (RF) positive patients. In the study of Lu *et al*. [9] no associations were found in the different RA subsets, these analyses were corrected for age and gender. We performed association analyses of the anti-CCP and RF patient subsets separately, for those patients with information available. We did not find an association between *LCE3C_LCE3B*-del and RA in any of the subsets analyzed (Table 2).

We performed an overall meta-analysis, using a recessive logistic regression model unadjusted for age and gender, on the combined data from this study and the studies of Docampo *et al.* [8] and Lu *et al.* [9] (Table 3). Despite the lack of significance of the association in the Dutch cohort, in the meta-analysis a significant association between *LCE3C_LCE3B*-del and RA was found (p<0.0001, OR 1.31 95%CI: 1.16–1.47). Next to the overall analysis, we performed also a meta-analysis on the anti-CCP and RF subgroups combining the Spanish, Chinese and our data (Table 3), unfortunately not for all patients these data were available. In this subgroup analysis all previously found associations [8] between *LCE3C_LCE3B*-del and RA were significant, with the association apparently strongest in RF positive subset of patients (p = 0.0007, OR 1.27, 95%CI: 1.11–1.45) (Table 3).

**Table 3:** Meta-analyses of association of the *LCE3C_LCE3B* deletion with RA in the Spanish, Chinese and Dutch cohort.

	Spain [8]					China [9]					Dutch					Meta-analyses					
	n	OR	L95	R95	p	n	OR	L95	R95	p	n	OR	L95	R95	p	n	OR	L95	R95	p	I^2^ (p value)
***LCE3C_LCE3B*** ** deletion**																					
all data	529	1.42	1.14	1.77	***0.001***	898	1.38	1.11	1.70	***0.0031***	1039	1.18	0.97	1.43	0.093	2466	1.31	1.16	1.47	***<0.0001***	0% (0.37)
CCP− subset	112	1.29	0.86	1.93	0.227	135	1.42	0.97	2.09	0.074	165	1.00	0.71	1.42	0.982	412	1.42	0.97	2.09	0.10	0% (0.39)
CCP+ subset	182	1.50	1.09	2.08	***0.012***	411	1.36	1.05	1.76	***0.021***	286	1.09	0.83	1.44	0.546	879	1.29	1.09	1.52	***0.002***	18% (0.30)
RF− subset	113	1.47	0.99	2.19	***0.008***	105	1.44	0.94	2.20	0.095	216	1.33	0.98	1.81	0.064	434	1.40	1.13	1.72	***0.002***	0% (0.92)
RF+ subset	360	1.40	1.09	1.79	***0.046***	382	1.37	1.05	1.78	***0.020***	736	1.14	0.92	1.40	0.231	1478	1.27	1.11	1.45	***0.0007***	0% (0.37)

Number of controls in the Spanish, Chinese and Dutch studies is 978, 681 and 779 respectively. The number of controls in the meta-analysis is 2438. *LCE3C_LCE3B* deletion: deletion of the *LCE3B* and *LCE3C* gene; del: deletion; CCP−: patients negative for anti cyclic citrullinated peptide; CCP+: patients positive for anti-cyclic citrullinated peptide; RF−: patients negative for rheumatoid factor; RF+ patients positive for rheumatoid factor. OR: odds ratio; L95: left border 95% confidence interval. R95: right border 95% confidence interval. I^2^ (p): measure for heterogeneity and p-value of the chi-square test for heterogeneity.

## Discussion

Although a previously reported association of the *LCE3C_LCE3B*-del with RA risk did not reach significance in our Dutch cohort, a meta-analysis combining our data and the previously reported Spanish and Chinese data [8], [9] confirmed the association of *LCE3C_LCE3B*-del with RA.

Our study had a power of over 95% to find an association, although we based this calculation on the previously found OR of 1.45. When we recalculate the power using the OR of the meta-analysis (OR = 1.31), the power of our study drops to 73%. A study population of 1256 samples will be needed to reach 80% power. The ORs observed in the Dutch population are in the same direction as seen in the previous studies [8], [9] and our meta-analysis showed a statistically significant association of the deletion with RA. This suggests that a larger population is necessary to prove whether the deletion is associated with RA. The contradictory findings from this study and the previous RA studies [8], [9] could also be caused by differences in the study population, e.g. disease phenotype or ethnicity. In addition, there is a discrepancy between the allele frequency of the deletion in the controls from Spain (55%) and China (54%) compared to The Netherlands (61%). This heterogeneity of *LCE3C_LCE3B*-del allele frequency in control groups from different ethnic backgrounds was already known from previous studies [6], [10]. Indeed, a recent meta-analysis confirming an association between *LCE3C_LCE3B*-del and psoriatic arthritis showed that the OR of the Spanish population was much higher than observed in an Italian population (1.66 versus 1.23) [10].

In our meta-analysis we used a recessive model, because this model fitted the *LCE* data in the psoriasis study best, based on the Akaike Information Criteria [11] which is in line with the two previous studies on *LCE3* in patients with RA [8], [9]. Although we could not identify an association between the deletion and RA in the Dutch cohort, addition of the data to the other studies resulted in lower p-values than without this dataset, showing a significant contribution of the Dutch population to the overall results. The addition of our Dutch samples leads to a lower OR and a smaller CI than previously reported. The other two studies may have significantly overestimated the magnitude of the odds ratio, the so-called winner's curse [12]. Due to publication bias it might be possible that some studies investigating the same subject have not been published, this information will be important to confirm the association between RA and the deletion as shown in this study.

In our subgroup analysis, we observed the strongest association in the RF positive patients. Larger datasets will be necessary to clarify whether the observed association is patient subset specific. In contrast to the results from our meta-analysis, genome-wide CNV analyses using the WTCCC RA dataset did not identify association with the *LCE3* region [4], [5]. One study only assessed rare CNVs (population frequency <5%) which excludes the *LCE3C_LCE3B*-del [5]. The other study included a probe covering the region that passed QC in their analysis (probe CNVR358.1, chr1:150,822,234–150,856,715 (UCSC genome browser hg18) [4]). It might be possible that the region is associated with RA in the WTCCC dataset at lower significance level than their genome-wide significance threshold.

Until now, *LCE3C_LCE3B-*del was found to be associated with psoriasis, psoriatic arthritis and RA in several populations [6], [7], [8], [9], [10], [11], [13], [14], whereas no association was found for atopic dermatitis [15]. Also in one study an association with systemic lupus erythematosus was found [9], implying that *LCE3C_LCE3B*-del may be a common risk factor for (auto)immune diseases. The function of the *LCE* genes has only been studied in skin in general [16], [17] and in relation to psoriasis [18]. From these studies it is known that the LCE proteins are likely to be incorporated in the cornified cell envelope, which is an important structure in the barrier function of skin. When comparing normal and psoriasis skin, the genes of the *LCE1, 2, 5* and *6* groups are mainly expressed in normal skin, whereas the *LCE3* genes are predominantly expressed in psoriasis skin. Moreover, upon barrier disruption of normal skin the *LCE1, 2, 5* and *6* gene-groups are downregulated, while the expression of the *LCE3* genes is upregulated. Altogether, these data imply a role in barrier repair for the LCE3 proteins and a role in barrier maintenance for the other LCEs. As hypothesized by Docampo *et al.* [8], the absence of *LCE3B* and *LCE3C* could compromise barrier function of the epithelia and possibly facilitate the entrance of environmental antigens or pathogens. Since the *LCE* genes are mainly expressed in skin and oral epithelia, these would be relevant tissues that could facilitate the entrance of antigens or pathogens, like the Epstein-Barr virus and the cytomegalovirus, thereby triggering RA. We reasoned that if this would be the case patients with *LCE3C_LCE3B*-del might be exposed more readily to common antigens and therefore would have an earlier age of onset due to easy access of pathogens/triggers through the skin. However, we did not observe such an association between the genotype and age of RA onset.

Our meta-analysis showed that the *LCE3B* and *LCE3C* deletion is associated with RA, though the contribution of our large Dutch sample is small. Therefore it will be necessary to test even larger patient cohorts to shed more light on the possible association of the deletion in specific RA patient subsets. An interesting next step would be to perform functional studies to unravel the mechanisms underlying this association.

## Materials and Methods

### Ethics statement

The ethical committee of the Radboud University Nijmegen Medical Centre (“Commissie Mensgebonden Onderzoek (CMO) Regio Arnhem Nijmegen”) approved the study (CMO number 2004/014); all patients and controls gave written informed consent.

### DNA samples

All patients met the American College of Rheumatology 1987 revised criteria for RA. The patients were part of the Dutch Rheumatoid Arthritis Monitoring registry (www.dreamregistry.nl) (n = 1039) [19], [20]. The controls (n = 779) were participating in the Nijmegen Biomedical Study [21]. For cohort characteristics see Table 1.

### Genotyping

DNA was extracted from blood using salt extraction. For the *LCE3C_LCE3B*-del genotyping a polymerase chain reaction was performed as earlier described [11]. 112 samples were genotyped in duplicate; error rate was 2.9% and assay failure rate was 0.3%. To validate the *LCE3C-LCE3B*-del genotype, we also genotyped the linked SNP rs4112788, which was performed using a Taqman® SNP genotyping assay (assay ID C__31910050_10) according to manufacturer's recommendations (Applied Biosystems, Nieuwerkerk aan den IJssel, The Netherlands). Five percent of the samples were analyzed in duplicate; all genotypes were concordant. The assay failure rate was 2.6%.

### Statistical analysis

Descriptive statistics for quantitative values are given as mean ± standard deviation (SD). Both variants showed no deviations from Hardy-Weinberg equilibrium for either patients or controls. Co-dominant and recessive logistic regression models were used to assess the genetic effect of *LCE3C_LCE3B*-del on RA risk, using homozygosity for the allele without deletion as a reference category. These models were similar to those used in the previous studies [8], [9]. The statistical analyses were performed using SPSS software 16.0 (SPSS Inc., Chicago, IL, U.S.A.). A meta-analysis was performed in Review Manager 5 (Review Manager (RevMan) Version 5.0. Copenhagen: The Nordic Cochrane Centre, The Cochrane Collaboration, 2008) using the aggregated data from this studies, Docampo *et al.* [8] and Lu *et al*. [9]. Heterogeneity of odds ratios (ORs) among cohorts was calculated using the Breslow-Day method, and pooled ORs were calculated under a fixed effects model (Mantel-Haenszel meta-analysis). A power calculation was performed using the Genetic Power Calculator [22] with input values derived from Docampo *et al.* [8] (significance level 0.05, OR = 1.45, allele frequency 0.62, recessive model).
